# Tofacitinib downregulates antiviral immune defence in keratinocytes and reduces T cell activation

**DOI:** 10.1186/s13075-021-02509-8

**Published:** 2021-05-21

**Authors:** Heike C. Hawerkamp, Alina Domdey, Lisa Radau, Philipp Sewerin, Péter Oláh, Bernhard Homey, Stephan Meller

**Affiliations:** 1 Department of Dermatology, Medical Faculty, Heinrich-Heine-University Düsseldorf, Moorenstr. 5, 40225 Düsseldorf, Germany; 2 Department and Hiller Research Unit for Rheumatology, Medical Faculty, Heinrich-Heine-University, Düsseldorf, Germany; 3 Department of Dermatology, Venereology and Oncodermatology, University of Pécs, Pécs, Hungary

**Keywords:** Janus kinase inhibitor, Antiviral immunity, Keratinocyte, T cell

## Abstract

**Background:**

Tofacitinib is a novel Janus kinase (JAK) inhibitor approved for the treatment of rheumatoid arthritis, psoriatic arthritis, and ulcerative colitis. In clinical trials, the most common adverse events observed were nasopharyngitis, upper respiratory tract infections, and zoster. JAKs are found downstream of the type II cytokine receptor family used by a number of T_H_17 cell-associated cytokines for signal transduction. These cytokines lead to the secretion of antiviral and antimicrobial peptides (AMPs) by keratinocytes or synoviocytes. Blocking the JAK pathway might result in a diminished secretion of antimicrobial and antiviral peptides causing higher susceptibility to infections in patients treated with JAK inhibitors.

**Methods:**

We treated primary human keratinocytes and synoviocytes with tofacitinib and subsequently added various cytokines and bacterial surface proteins before evaluation of the response via RT-qPCR. CD69 expression on tofacitinib-treated PBMCs was investigated via flow cytometry.

**Results:**

We found a markedly reduced gene expression of all tested antiviral peptides such as MX1 or ISG15 in keratinocytes and synoviocytes in the presence of tofacitinib in vitro. Additionally, we found that JAK inhibition reduced activation of T cells after stimulation with bacterial LPS or viral VZV gE.

**Conclusions:**

The antiviral immunity is strongly inhibited in the presence of tofacitinib in vitro, while the antimicrobial immunity does not seem to be affected. In T cells, the overall activation process seems to be influenced by tofacitinib. These findings suggest that tofacitinib has an impact on antiviral immunity such as patients treated with tofacitinib often show adverse events like herpes zoster.

**Graphical abstract:**

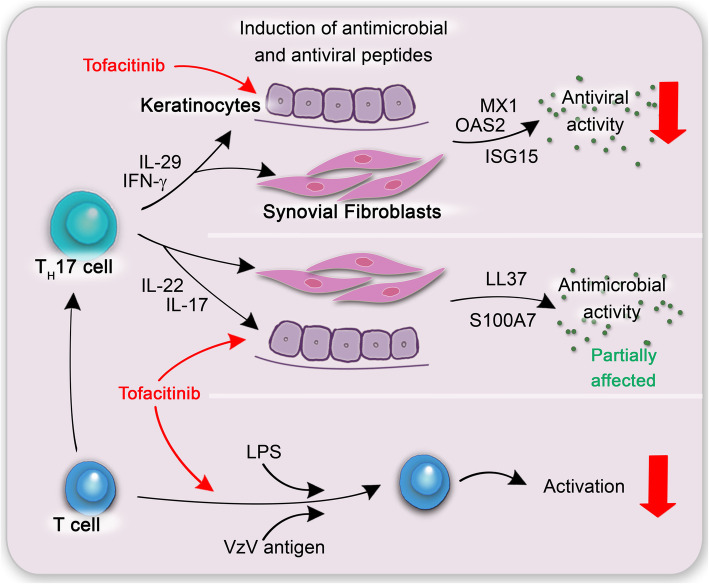

## Introduction

Tofacitinib is an oral Janus kinase inhibitor used in the treatment of severe rheumatoid arthritis (RA), psoriasis arthritis (PsA), and ulcerative colitis [1–3]. This JAK inhibitor is most efficient in inhibiting JAK1 and JAK3 signalling pathways, but a partial efficiency on JAK2 pathways is also seen [2, 4, 5].

RA is a chronic autoimmune disease characterized by a destruction of the joint accompanied by stiffness and inflammation as well as pain [2]. The thickness of the synovium is increasing about 10-fold due to increased proliferation of synoviocytes [6–8]. Synoviocytes can be divided into type A synoviocytes and type B synoviocytes, which are also called human fibroblast-like synoviocytes (HFLS) [7, 8]. HFLS secrete a variety of proteins important for the extracellular matrix but also cytokines are in their repertoire such as interleukin (IL)-6 or tumour necrosis factor (TNF)-α [6, 7]. The efficiency of tofacitinib in RA can partly be explained by the underlying high expression of JAK3 and the STATs 1, 4, and 6 in the synovium of the joints of RA patients [9]. PsA is occurring in about 10 to 42% of patients that already suffer from plaque psoriasis [10–12]. The whole architecture of the outer layers of skin is based on the well-organized proliferation and differentiation of keratinocytes [13].

Keratinocytes protect the human body from exogen pathogens by producing antimicrobial peptides (AMPs) and antiviral peptides (AVPs) but also (pro-) inflammatory chemokines and cytokines [14, 15]. AVPs target various steps in the virus life cycle, e.g. inhibiting the entry of the virus into the host cell [16]. AVPs are induced by the virus itself but also by the T_H_17 cell-derived IL-29 (also called IFN-λ1) has been shown to induce the production of AVPs such as MX1, OAS2, BST2, and ISG15 in keratinocytes [17]. The main classes of AMPs in humans are cathelidins and defensins [18, 19]. AMPs bind to bacterial components such as lipopolysaccharides (LPS) or lipoteichoic acids (LTA). In the further process, these AMPs integrate themselves into the membrane of the bacteria and form holes or prevent the bacteria from division due to the disrupted membrane [20–24]. Among other factors, T_H_17-associated cytokines induce the secretion of AVPs as well as AMPs by both, keratinocytes and synoviocytes, respectively. A number of cytokines that signal via type I and type II receptors are secreted by T_H_17 cells that belong to the effector T cells [25]. JAKs are found downstream of the type I and type II cytokine receptors. Binding of the respective cytokine to its receptor then leads to the activation of the JAK signalling pathway via a phosphorylation of signal transducers and activator of transcription (STAT) [5].

The T_H_17 cytokines IL-22, IL-26, and IL-29 bind to receptors that belong to the type II cytokine receptor family [26]. The binding of these interleukins to their respective receptor heterodimer on their target cells such as keratinocytes or synoviocytes induces a signal transduction via the JAK/STAT pathway. Overall, a number of cytokines produced by T_H_17 cells are leading to the secretion of AVPs and AMPs by keratinocytes or synoviocytes. These cytokines stimulate their target cells via the type II cytokine receptor family and these receptors use the JAK pathway for signal transduction. Blockage of the JAK pathway might therefore result in a diminished secretion of AVPs and AMPs leading to a higher susceptibility to infections. In fact, nasopharyngitis, upper respiratory tract infections, and herpes zoster were among the most common adverse events observed in clinical trials investigating the therapeutical effects of tofacitinib [27, 28]. Interestingly, the incidence of herpes zoster under tofacitinib treatment is dose dependent [29].

Additionally, also IL-6 and IL-23, cytokines involved in the differentiation of T_H_17 cells, do also signal via the JAK/STAT pathway [30, 31]. T_H_17 cells have long been thought to be highly pathogenic but more and more publications provide data and evidence on non-pathogenic T_H_17 cells or subtypes of T_H_17 cells with regulatory functions [32, 33]. Lee and colleagues have demonstrated that the pathogenicity of T_H_17 cells is dependent on the cytokines present [34]. They found that T_H_17 cells generated in the presence of only transforming growth factor (TGF)-β1 and IL-6, but not IL-23, do not induce autoimmune diseases. A blockage of the JAK pathway might also influence the T cell differentiation pattern which means that a JAK inhibition could drive the differentiation of T_H_17 cells towards a more regulated phenotype instead of a pathogenic phenotype [35].

Here, we investigated in vitro the effects of tofacitinib on epithelial cells such as keratinocytes and synoviocytes as important players in PsA and RA, respectively. Keratinocytes are furthermore target cells when reactivated varicella zoster leads to herpes zoster. Additionally, we aim to shed light on the role of JAK inhibition on TH_H_17 cell development. We report that antimicrobial immunity does not seem to be affected by tofacitinib, but the antiviral immunity is strongly inhibited in the presence of tofacitinib in vitro.

## Material and methods

### Human subjects

Buffy Coats from healthy individuals were obtained from the Institute of Haemostasis and Transfusion Medicine, University Hospital Düsseldorf. For experiments involving varicella antigens, blood in four BD Vacutainer Cell Preparation Tubes (CPT™, BD Biosciences) was taken from healthy volunteers. The guidelines of the Declaration of Helsinki were deployed, and the study was authorized by the local ethics committee (No: 5775R).

### Normal primary human keratinocytes

Normal primary human keratinocytes were isolated from the foreskin or skin derived from mamma reduction or other surgeries. The keratinocytes were seeded in 12-well plates and cultured until sub-confluence. Tofacitinib (100 nM or 600 nM) (Sigma Aldrich, USA) was added to the respective well and incubated for 60 min at 37 °C before addition of 1 μg/ml LPS or LTA (InvivoGen, France) or 100 ng/ml of all the interleukins (IL-17, IL-22, IL-29) and interferon gamma (IFN-γ) (R&D Systems, USA). After 24 h of incubation, the supernatants were collected, and the keratinocytes were harvested and lysed for subsequent RNA isolation.

### Primary human fibroblast-like synoviocytes

Primary human fibroblast-like synoviocytes (HFLS) from normal human healthy synovial tissue were obtained from Cell Applications Inc. (San Diego, USA). The cells were maintained in synoviocyte growth medium (ready-to-use, Cell Applications) or DMEM GlutaMAX (Gibco) supplemented with 10% foetal calf serum (FCS) and 1% penicillin/streptomycin. Stimulation conditions were analogous to keratinocytes in 12-well plates and the cells as well as supernatant were harvested after 24 h.

### Isolation of peripheral blood mononuclear cells

Peripheral blood mononuclear cells (PBMCs) were isolated from Buffy Coats using standard Ficoll centrifugation.

### RNA isolation, cDNA synthesis, and qPCR

RNA isolation was performed using the RNeasy Mini Kit (Qiagen, Netherlands) according to the manufacturer’s instructions. Transcription into cDNA and subsequent qPCR was conducted as previously described [36].

### QPCR primers

Used qPCR primers were the following: S100A7 (reverse: 5′ TGT CCT TTT TCT CAA AGA CGT C 3′, forward: 5′ AGA CGT GAT GAC AAG ATT GAC 3′), MX1 (reverse: 5′ TTC TTC CAG CTC CTT CTC TCT G 3′, forward: 5′ AGA GAA GGT GAG AAG CTG ATC C 3′), ISG15 (reverse: 5′ CCA GCA TCT TCA CCG TCA G 3′, forward: 5′ GCG AAC TCA TCT TTG CCA GTA 3′), OAS2 (reverse: 5′ TTC CTG GTG TCT GCA TTG TC 3′, forward: 5′ CTG GCA AAA GAA GCA AAG GA 3′), IFNG (reverse: 5′ GTT CCA TTA TCC GCT ACA TCT GAA 3′, forward: 5′ ACG TCT GCA TCG TTT TGG GTT 3′), TBET (reverse: 5′ TGG TCT ATT TTT AGC TGG GTG ATG TCT G 3′, forward: 5′ GGT GGT AAC ATG CCA GGG AAC AGG A 3′), RORC (reverse: 5′ CGG AAG AAG CCC TTG CAC CCC 3′, forward: 5′ GAC AGC ACC GAG CCT CAC GG 3′), and GATA3 (reverse: 5′ GGG GCC GGT TCT GTC CGT TC 3′, forward: 5′ CCG GTC CAG CAC AGA AGG CA 3′). Additionally, TaqMan Gene Expression Assays (ThermoFisher Scientific, Waltham, USA) such as CAMP (Hs01011708_m1), GMCSF (Hs00171266_m1), IL17A (Hs99999082_m1), and IL22 (Hs00220924_m1) were performed. As endogenous control, the 18S rRNA primer-probe set from ThermoFisher Scientific was used.

### T cell generation and stimulation

Naïve T cells were isolated from the PBMCs using the Naïve CD4^+^ T Cell Isolation Kit II (Miltenyi Biotec, Bergisch Gladbach, Germany) according to the manufacturer’s instructions. Subsequently, the naïve T cells were activated with anti-CD2/anti-CD3/anti-CD28 beads to mimic antigen-presenting cells by using the Human T Cell Activation/Expansion Kit (Miltenyi Biotec). Activated T cells were then seeded at a concentration of 2.5 × 10^6^ cells per ml into a 24-well plate. Then, tofacitinib was added at concentrations of 100 nM or 600 nM for 30 min before TGF-β1 (20 ng/ml), IL-6 (20 ng/ml), IL-23 (10 ng/ml), and anti-IL4 (1 μg/ml) were added and incubated for 3 days. At day 3, the cell clumps were homogenized, and the cell suspension was split and supplemented with 5 ng/ml IL-2. Cell analysis was done after another 4 days of incubation.

### Flow cytometry of polarized T cells

T cells polarized towards T_H_17 subtype in the presence or absence of tofacitinib were analyzed via flow cytometry. In order to investigate intracellular cytokines such as IL-17 or IL-26, Brefeldin A (Biolegend, San Diego, USA) and GolgiStop (BD Biosciences, Franklin Lakes, USA) were added to the cells 4 h prior to the incubation end. The T cells were then suspended and centrifuged for 5 min at 1500 rpm. The cell pellet was suspended in 500 μl flow cytometry (FACS) buffer (PBS with 4% FCS and 1 mM EDTA). Then, 25% human AB serum was added to the samples and incubated for 15 min on ice before another 500 μl FACS buffer was added and the samples centrifuged at 1500 rpm for 5 min. The cells were then stained with antibodies against surface molecules such as CD8 (PE, clone: RPA-T8, BD Pharmingen), CD4 (PE-Cy7, clone: OKT4, BioLegend), and Zombie NIR (APC-Cy7; life/dead marker, BioLegend) in a volume of 50 μl for 30 min on ice. After the incubation, 500 μl FACS Buffer was appended and the samples centrifuged as described before. The cells were suspended in 100 μl of a 4% paraformaldehyde (PFA) solution and incubated at 4 °C for 15 min. The cell pellet was then suspended in 200 μl FACS buffer before intracellular cytokine staining was implemented. The cells were permeabilized by using 1000 μl of Perm/Wash solution (BD Biosciences) per sample and incubated for 15 min at RT. Antibodies against IL-17 (PerCP, clone: 41802, Biotechne) and IL-26 (APC, clone: 11C31, US Biological, Salem, USA) were then diluted in Perm/Wash solution, added to the cells at a final volume of 50 μl, and incubated for 30 min on ice. Thereafter, 500 μl Perm/Wash solution was added and the samples were centrifuged. Finally, the cells were suspended in 200 μl FACS buffer and analysed on a CytoFlex S flow cytometer (Beckman Coulter, Brea, USA) together with CytExpert software.

### Flow cytometry of varicella-antigen stimulated PBMCs

Four CPT blood tubes were taken from healthy volunteers and centrifuged for 20 min at 2500 rpm at RT. After centrifugation, the PBMCs are transferred into a Falcon tube. The PBMCs were then washed with PBS and red blood cells were lysed in 10 ml ACK lysis buffer for 10 min at 4 °C. The lysis reaction was stopped by an excess amount of PBS after the cells were then pelleted again via centrifugation for 10 min at 1250 rpm. The cells were then counted and adjusted to a concentration of 5 × 10^6^ cells/ml in RPMI1640 medium (Gibco) supplemented with 5% human AB serum. Then, 200 μl (1 × 10^6^ cells) was transferred into a 1.5-ml Eppendorf tube per condition. Tofacitinib at final concentrations of 100 or 600 nM was added to the respective tube and incubated for 45 min at 37 °C. Now varicella zoster virus envelope glycoprotein E (VZV gE) (PepMix, JPT solutions, Berlin, Germany) was added at a final concentration of 10 μg/ml to respective tubes. Furthermore, lipopolysaccharide (LPS, 100 ng/ml), DMSO, and anti-CD2/anti-CD2R antibodies (BD FastImmune™, BD Biosciences) were added to the respective tubes and served as controls. The tubes were incubated for 20 h at 37 °C followed by flow staining. The cells were proceeded as described above and stained with antibodies against the surface markers CD3 (APC, clone: UCHT1, BioLegend) and CD69 (PE, clone: FN50, BioLegend) together with the life/dead marker Zombie NIR (APC-Cy7). Isotype controls (IgG1) were included as well.

### MicroArray

RNA from untreated keratinocytes or keratinocytes treated with 600 nM tofacitinib were subjected to a DNA MicroArray (*Affymetrix human PrimeView 2.0*)*.* Prior to the MicroArray, the RNA quality was evaluated at the Biomedical Research Centre (Biomedizinisches Forschungszentrum (BMFZ)) at the Heinrich-Heine-University, Düsseldorf.

### Cell viability assays

Cell viability of keratinocytes was evaluated using the 3-(4,5-dimethylthiazol-2-yl)-2,5-diphenyltetrazolium (MTT) assay. Keratinocytes were seeded into a flat-bottom 96-well plate and grown until sub-confluence. Then, the cells were stimulated with a serial dilution of tofacitinib (10 to 0.05 μM) and DMSO as diluent control for 24 h at 37 °C. To the untreated control, cell culture media were added and the negative control was 5% DMSO. After the addition of 5 mg/ml MTT and an incubation for 2 h, the reaction was stopped, and the OD was measured at 540 nm. The OD values were converted into percentages with the untreated control set to 100%.

For the cell viability of both synoviocytes and T cells, the CellTiter-Glo® Luminescent Cell Viability Assay (Promega) was used according to the manufacturer’s instructions. T cells and synoviocytes were stimulated analogously to keratinocytes for 24 h at 37 °C. For both those cell types, 1% Triton served as the negative control. After the incubation time, CellTiter-Glo® Buffer was added CellTiter-Glo® Substrate to generate CellTiter-Glo® Reagent. One hundred microliters of CellTiter-Glo® Reagent was then added to each well, and the luminescence was measured immediately on a TECAN infinite M200 Pro plate reader.

### Statistical analysis

All analyses were done using GraphPad Prism version 5.03 (GraphPad Software, Inc.). The Mann-Whitney *U* test or Wilcoxon matched pairs signed rank test was used to calculate statistical significances which were then depicted as follows: “*” equals *P* ≤ 0.05, “**” equals *P* ≤ 0.01, and “***” equals *P* ≤ 0.001.

## Results

### Tofacitinib affects predominantly the expression of antiviral peptides while antimicrobial peptides are largely unaffected

**Fig. 1:**
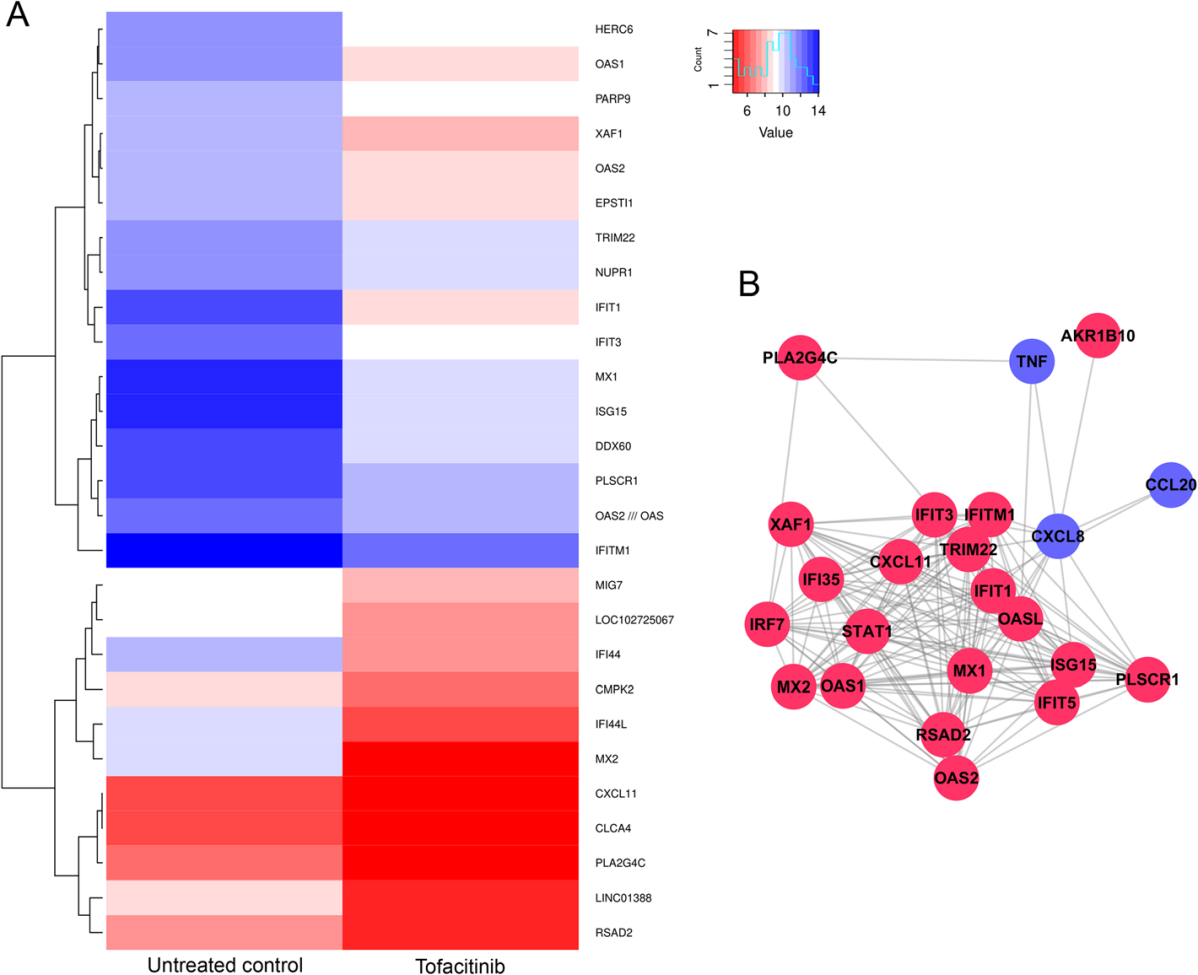
Tofacitinib strongly downregulates antiviral peptides but only minimally affects gene expression of antimicrobial peptides. **a** Keratinocytes were stimulated with tofacitinib (600 nM) or left untreated (control). The cells were harvested after 24 h; RNA was isolated from cell lysates and used for DNA MicroArray. Blue colour indicates upregulated genes, while red colour indicates downregulated genes compared to baseline. **b** Network analysis focusing on antimicrobial and antiviral genes

Initially, we tested the cell viability of keratinocytes, synoviocytes, and T cells in the presence of tofacitinib in a range of concentrations (Supplemental Fig. 1). After confirming that cell viability is not affected by tofacitinib, we subjected RNA from keratinocytes treated with 600 nM tofacitinib or untreated to DNA MicroArray. To display a broad overview of differentially regulated genes, a heatmap is shown in Fig. 1a. The presence of tofacitinib quite drastically changes the overall gene expression pattern. Blue depicts upregulated genes, while red shows downregulated genes. A number of antiviral genes are downregulated (such as MX1, MX2, OAS1), while antimicrobial genes such as S100A8 and S100A9 (lower end of heat map) seem to only be minimally affected by JAK inhibition (Fig. 1a). Focusing only on antimicrobial and antiviral gene expression, a network graphic was generated based on the GO term annotations (Fig. 1b).

We next investigated the impact of JAK inhibition on the production of antimicrobial peptides psoriasin (S100A7), and the cathelicidin antimicrobial peptide (CAMP, also called LL37) by keratinocytes and synoviocytes after stimulation with bacterial components and cytokines in a larger cohort (Supplemental Fig. 2 and 3). We stimulated the cells with LPS or LTA (Supplemental Fig. 2b, f). Additionally, the following cytokines were used: IL-17, IL-22 (Supplemental Fig. 2c, g), and IL-29, as well as the antiviral interferon (IFN)-γ (Supplemental Fig. 2d, h). As expected, the expression of CAMP in keratinocytes is slightly induced by LTA and IL-29 and potently enhanced by IL-17. Tofacitinib alone does not affect CAMP expression negatively (Supplemental Fig. 2). Synoviocytes responded to treatment with LPS with significant increased CAMP expression (Supplemental Fig. 2f). This increase was efficiently reduced by tofacitinib (600 nM) (Supplemental Fig. 2f).

All bacterial components or cytokines, except IFN-γ, induced S100A7 expression in keratinocytes (Supplemental Fig. 3). We found that the JAK inhibition using tofacitinib minimally affects the gene expression of psoriasin (S100A7) in keratinocytes (Supplemental Fig. 3a). Looking at the keratinocytes treated with IL-22 and tofacitinib, we see a trend towards downregulation of S100A7 expression in the presence of tofacitinib compared to keratinocytes treated with IL-22 alone (Supplemental Fig. 3c). Investigating other S100 proteins such as S100A8 and S100A9, we found a similar gene expression pattern as for S100A7 keratinocytes (data not shown). An effect of JAK inhibition on the synovial expression of psoriasin (S100A7) could not be detected (Supplemental Fig. 3e-h).

**Fig. 2:**
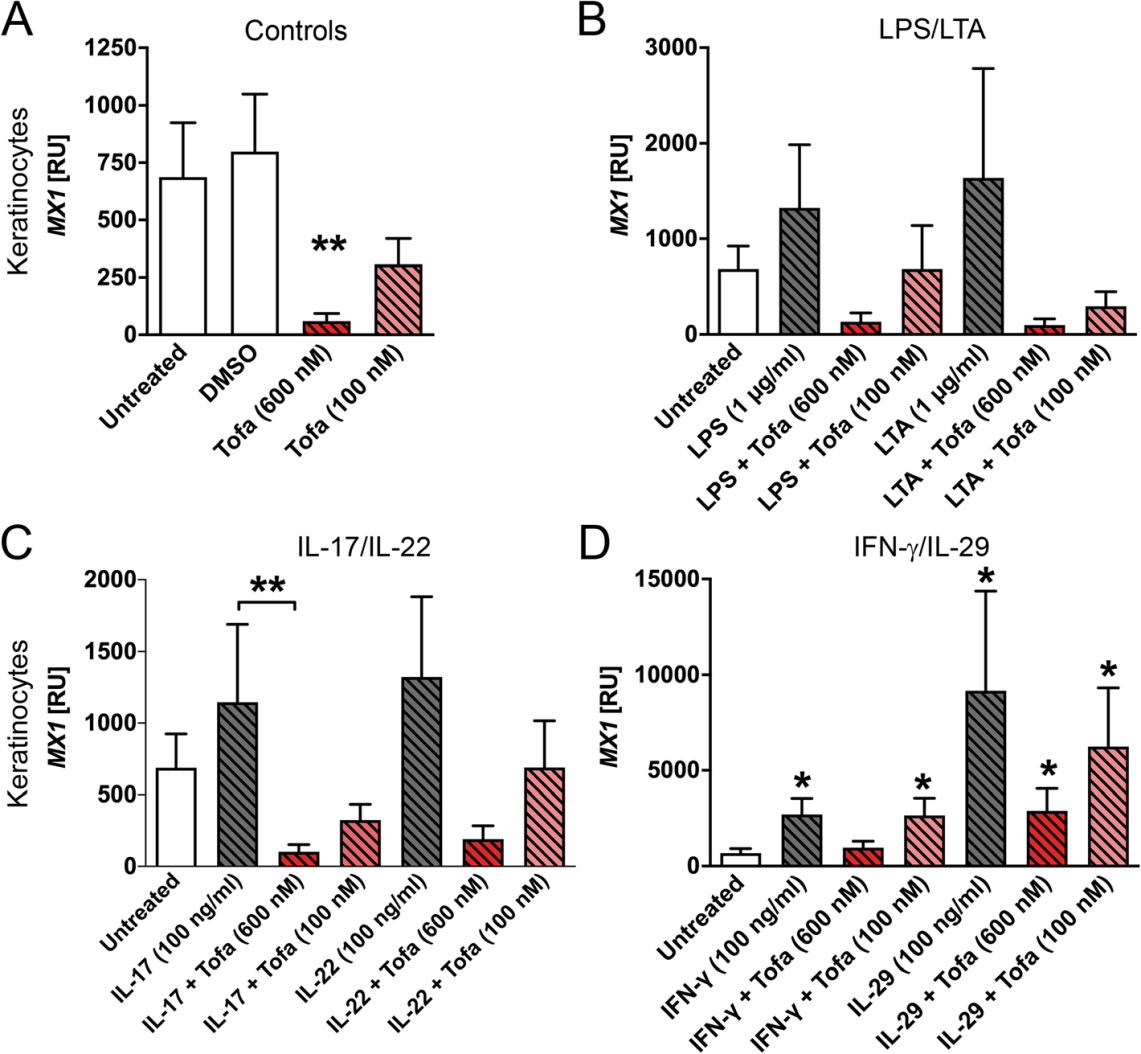
Tofacitinib downregulates gene expression of MX1 at baseline or in the presence of IFN-γ or IL-29. Keratinocytes (*n* = 5–8) were stimulated for 60 min with tofacitinib before addition of the respective cytokine or bacterial component. MX gene expression is shown for controls (**a**), tofacitinib in combination with LPS or LTA (**b**), in combination with IL-17 or IL-22 (**c**), or tofacitinib combined with IFN-γ or IL-29 (**d**). The cell lysates were analysed via qPCR. Statistical calculation was done using the Mann-Whitney *U* test. Significances that were compared to the untreated control are depicted directly above the stimulatory agent or the compared conditions are indicated by lines and depicted as follows: *P ≤ 0.05, **P ≤ 0.01, and ***P ≤ 0.001

After AMPs, we focused on AVPs starting with MX1 and interferon-stimulated gene 15 (ISG15). Interestingly, MX1 is induced in keratinocytes by all added bacterial components and cytokines (Fig. 2a–d). The highest induction of MX1 is seen when 100 ng/ml IL-29 is added to keratinocytes (Fig. 2d). Tofacitinib alone drastically downregulates MX1 expression at 600 nM (Fig. 2a). In synoviocytes, we found that MX1 is significantly downregulated in the presence of tofacitinib (600 nM) alone (Supplemental Fig. 4a). Of note, LPS (1 μg/ml) induced a significant increase of MX1 gene expression (Supplemental Fig. 4b). This increase is again reduced when the cells were treated with tofacitinib (600 nM). As expected also, IFN-γ and IL-29 enhance MX1 gene expression in synoviocytes (Supplemental Fig. 4d). Again, tofacitinib inhibits this upregulation. When comparing the IL-29 induced MX1 expression to the expression after treatment with both tofacitinib and IL-29, a significant reduction was observed (Supplemental Fig. 4d).

**Fig. 3:**
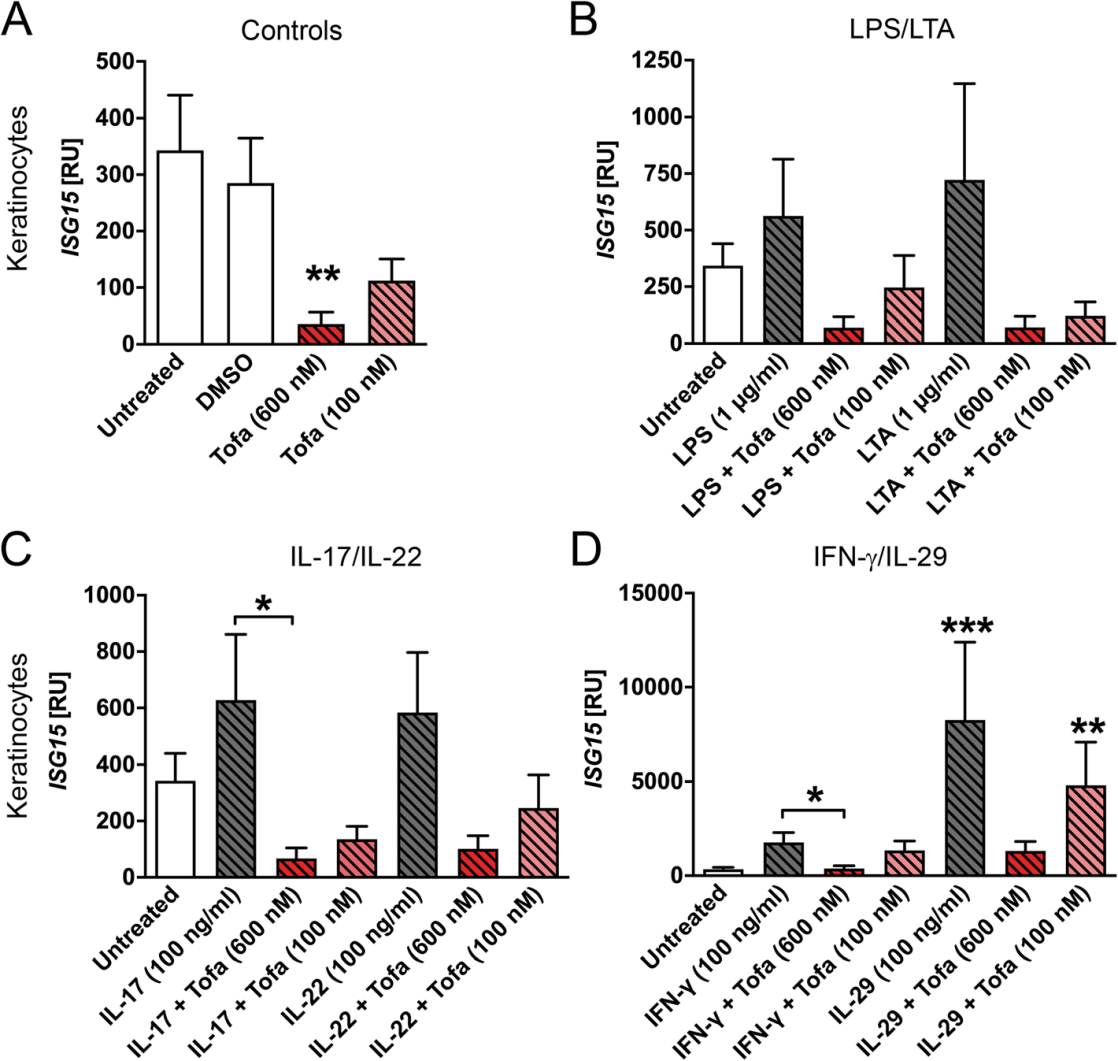
Antiviral ISG15 gene expression is downregulated by tofacitinib at the baseline level. Keratinocytes (*n* = 5–8) were stimulated for 60 min with tofacitinib before addition of the respective cytokine (IL-17, IL-22, IFN-γ, or IL-29) or bacterial component (LPS or LTA). The cell lysates were analysed via qPCR. Statistical calculation was done using the Mann-Whitney *U* test. Significances that were compared to the untreated control are depicted directly above the stimulatory agent or the compared conditions are indicated by lines and depicted as follows: *P ≤ 0.05, **P ≤ 0.01, and ***P ≤ 0.001

For ISG15, there is also an induction with all bacterial components and cytokines in keratinocytes (Fig. 3). Similar to MX1, tofacitinib is pronouncedly inhibiting ISG15 expressions (Fig. 3a to d). Also, in synoviocytes, the pattern of ISG15 gene expression is very similar to the one of MX1 (Supplemental Fig. 5a to d). In contrast, here, the downregulation of ISG15 gene as the baseline level did not reach significance (Fig. 3a). For ISG15, we further found that tofacitinib even at the low concentration of 100 nM significantly inhibited IL-29 induced ISG15 expression in synoviocytes (Supplemental Fig. 5d).

**Fig. 4:**
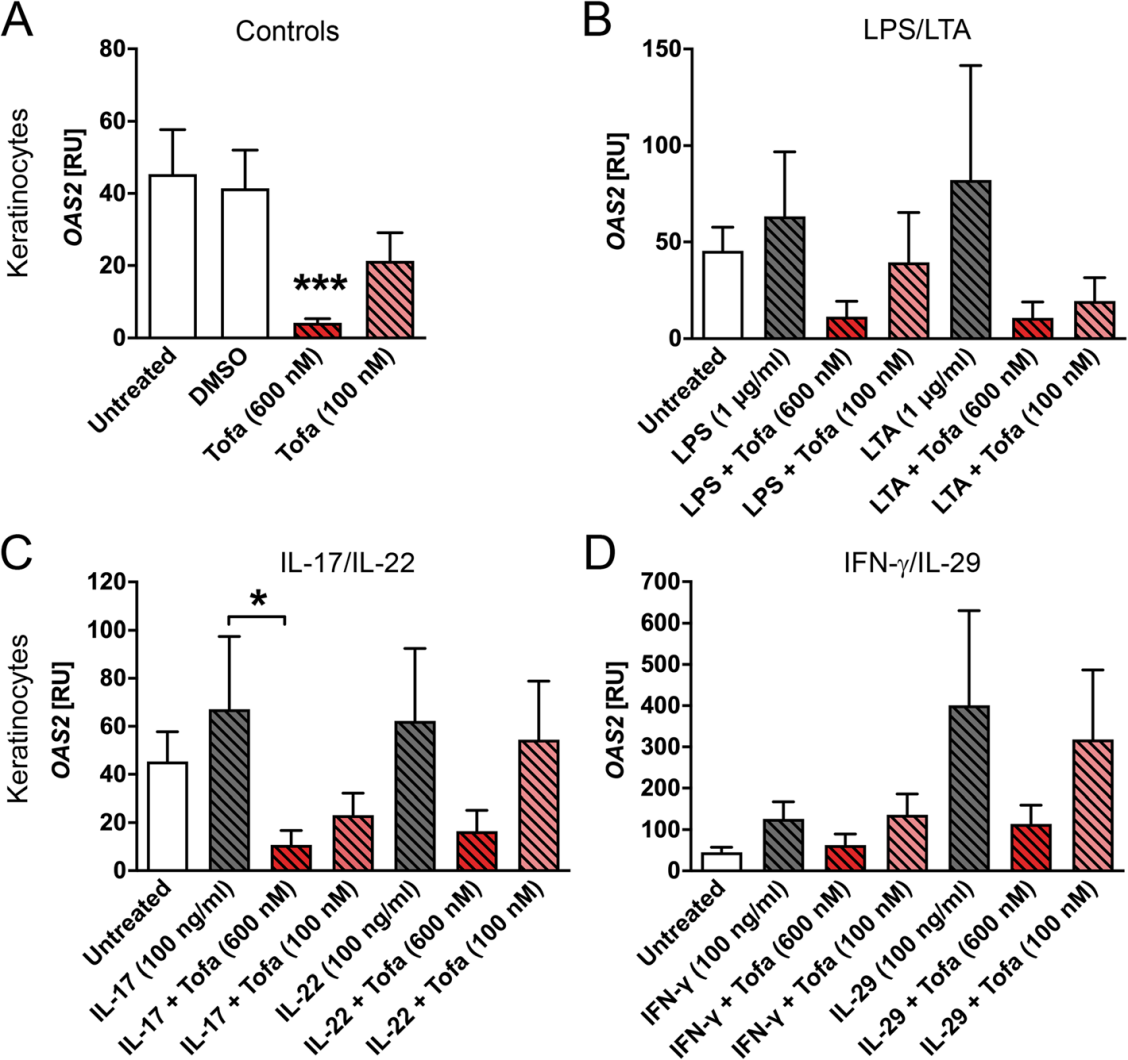
Tofacitinib downregulates gene expression of antiviral OAS2. Keratinocytes (*n* = 5–8) were stimulated for 60 min with tofacitinib before addition of the respective cytokine or bacterial component. OAS2 gene expression is shown for controls (**a**), tofacitinib in combination with LPS or LTA (**b**), in combination with IL-17 or IL-22 (**c**), or tofacitinib combined with IFN-γ or IL-29 (**d**). The gene expression was analysed via qPCR. Statistical analysis was done using the Mann-Whitney *U* test. Significances that were compared to untreated control are depicted directly above the stimulatory agent or the compared conditions are indicated by lines and depicted as follows: *P ≤ 0.05 and ***P ≤ 0.001

We then investigated the gene expression of 2′-5′-oligoadenylate synthetase 2 (OAS2) and found that OAS2 is induced by all co-stimulatory agents added, and even highly induced in the presence of IFN-γ or IL-29 (Fig. 4). The inhibition of the JAK pathway markedly reduced OAS2 gene expression (Fig. 4a and Supplemental Fig. 6a). Interestingly, synoviocytes responded to LPS treatment with significantly increased expression of the antiviral peptide OAS2 (Supplemental Fig. 6b). The pre-treatment with tofacitinib before the addition of LPS led to a strong trend in the reduction of OAS2 expression (Supplemental Fig. 6b). Similar to ISG15, also OAS2 expression was induced by IFN-γ and IL-29. Additionally, the OAS2 expression was reduced when tofacitinib was co-incubated with IL-29 (Supplemental Fig. 6d). In the presence of IFN-γ, the OAS2 expression is not modified by JAK inhibition (Supplemental Fig. 6d).

Overall, AMPs from keratinocytes and synoviocytes are barely affected by the presence of tofacitinib. This is in great contrast to AVPs which are efficiently downregulated or completely inhibited by JAK inhibition in those two cell types.

### Effect of JAK inhibition of T cell differentiation

We aimed to elucidate if the presence of tofacitinib drives the T_H_17 cell differentiation to a more classical or a more pathogenic phenotype. Naïve CD4^+^ T cells were differentiated towards T_H_17 cells using IL-6, IL-23, TGF-β, anti-IL-4, and anti-IFN-γ in the presence or absence of tofacitinib. Both gene expressions and proteins were analysed at day 7 of culture. We found that tofacitinib at both tested concentrations of 600 nM and 100 nM slightly upregulates RORC, a transcription factor mainly for classical T_H_17 cells (Supplemental Fig. 7a). The expression of TBET, which is more characteristic for pathogenic T_H_17 cells, is only slightly affected by the presence of 600 nM tofacitinib (Supplemental Fig. 7d). As a control gene, the expression of GATA3 was analysed, which is a transcription factor important for T_H_2 cells and which was not affected by the presence of tofacitinib (data not shown). Considering the gene expression of relevant cytokines, we saw a slightly increased expression of IL17A in the presence of tofacitinib (Supplemental Fig. 7b). IFNG gene expression seems only to be increased by 100 nM tofacitinib (Supplemental Fig. 7e). Considering IL22 gene expression, no change with tofacitinib was observed (Supplemental Fig. 7b). A similar finding is seen for GMSCF (Supplemental Fig. 7e). The following protein expression analysis revealed a trend towards upregulated IL-17 in CD4^+^ T cells under JAK inhibition (Supplemental Fig. 7c). Additionally, we investigated the T_H_17 cell-derived IL-26, an interleukin with antimicrobial properties, and saw a significant downregulation of IL-26 in T cells treated with both 100 nM and 600 nM tofacitinib (Supplemental Fig. 7c).

### Tofacitinib inhibits T cell activation response to varicella zoster virus and LPS

**Fig. 5:**
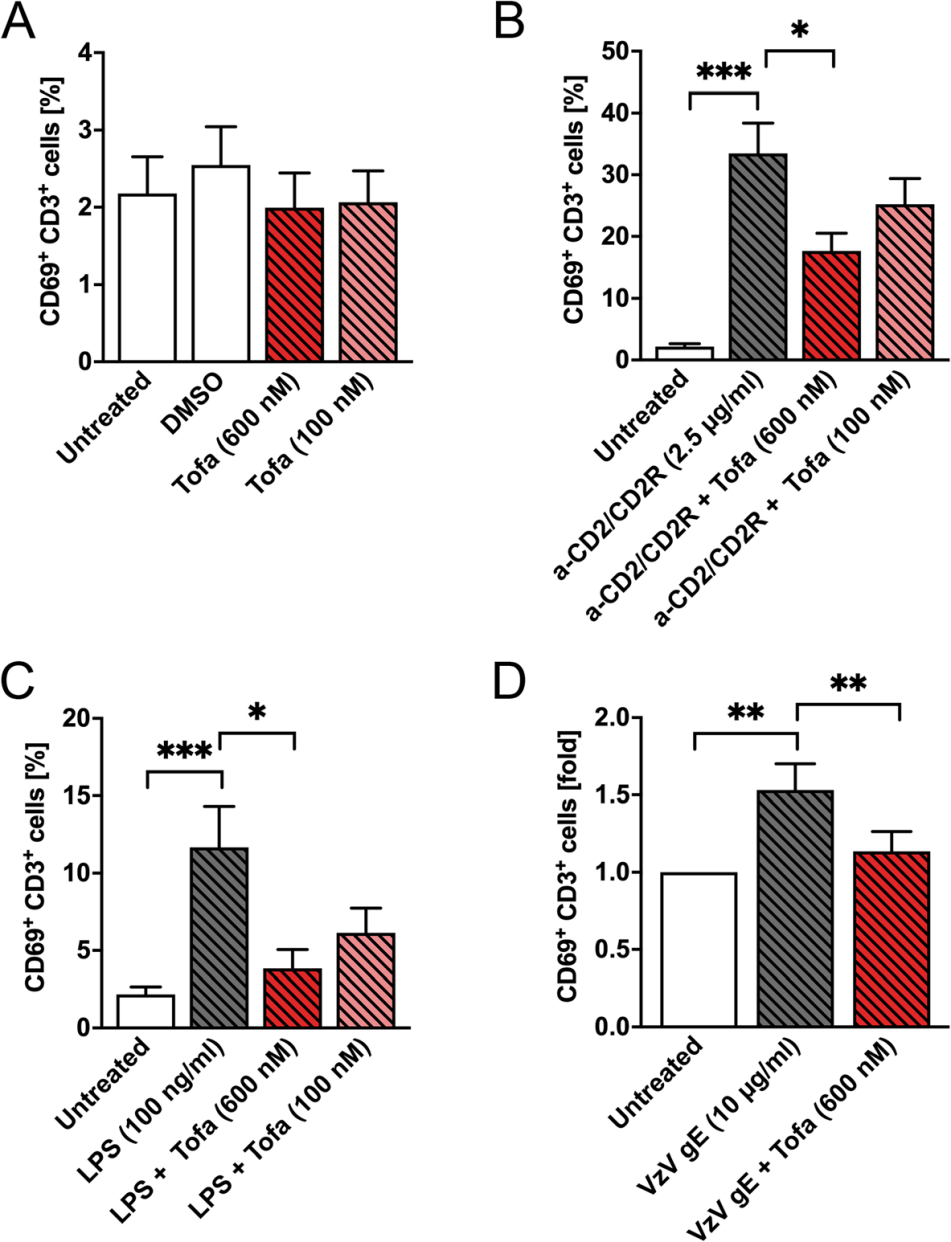
Tofacitinib inhibits T cell activation. Peripheral blood mononuclear cells (PBMCs) from healthy donors (*n* = 9) were pre-incubated with tofacitinib (100 or 600 nM) for 45 min before the addition of LPS or varicella zoster virus envelope glycoprotein (VZV gE) for another 23 h. An antibody against CD2/CD2R was used as a positive control. The cells were then stained with antibodies against CD69 and CD3 surface markers. A life/dead marker was included to analyse viable cells only. The cells were analysed via flow cytometry and the percentage of CD69^+^CD3^+^ cells was depicted for the positive controls and LPS; for VZV gE, the CD69^+^CD3^+^ cells were displayed as fold increase compared to the untreated control. Statistical significance was calculated using the Mann-Whitney test for the data displayed in percent while Wilcoxon matched pairs signed rank test was applied when fold inductions as displayed. Statistical significances are indicated as *P ≤ 0.05, **P ≤ 0.01, and ***P ≤ 0.001

Increased varicella zoster infections are a main adverse event in the treatment of patients with tofacitinib. To investigate if tofacitinib directly affects cellular response to antigens from varicella zoster virus (VZV), we pre-treated PBMCs from healthy donors with tofacitinib (100 and 600 nM) before the addition of LPS or VZV envelope glycoprotein (VZV gE). The response was then measured via flow cytometry where the expression of the surface activation marker CD69 was investigated. We found that CD69 expression is not altered at the baseline level by the presence of tofacitinib (Fig. 5a). The anti-CD2/CD2R antibody (positive control) significantly induced CD69 expression on CD3^+^ T cells as expected (Fig. 5b). The presence of tofacitinib at the high concentration of 600 nM significantly downregulates the anti-CD2/CD2R-induced CD69 expression (Fig. 5b). Both LPS and VZV gE alone induced a significant increase in CD69 surface expression, which was completely blocked by the presence of tofacitinib (Fig. 5c, d).

In summary, T cell activation is inhibited by a blockade of the JAK pathway using tofacitinib. This inhibition appears to be independent of any specific activation signalling cascade.

## Discussion

Treatment with tofacitinib goes along with increased incidences of infections. Against this background, we investigated the effects of this agent on the expression of AVP and AMP as well on T cell differentiation and activation.

AVPs are not only induced by the virus itself. The T_H_17 cell-derived IL-29 (also called IFN-λ1) has been shown to induce the production of AVPs such as MX1, OAS2, BST2, and ISG15 in keratinocytes [17]. For RA, an overexpression of IL-29 was shown and furthermore the IL-29 induced expression of proinflammatory cytokines such as IL-6, but also IL-8 in RA fibroblast-like synoviocytes [37, 38]. In fact, gene expression of all tested AVPs such as MX1 is markedly reduced in a dose-dependent manner even without any co-stimulatory cytokine or bacterial component added. The induction of AVPs, e.g. by IFN-γ or IL-29, is strongly inhibited by the presence of tofacitinib in keratinocytes. As both IFN-γ and IL-29 [39] use the JAK-STAT pathway for signal transduction, tofacitinib most likely does not only inhibit gene expression but the signal is not even transmitted into the cell. Our data on MX1 and ISGs are in line with the literature where splenocytes from lupus-prone mice treated with tofacitinib (10 mg/kg daily) showed also decreased expression of MX1 and ISGs in comparison to untreated control animals [40]. Our results indicate an important direct effect of tofacitinib on keratinocytes and thus should be considered in the systemic treatment of psoriasis arthritis. Considering synoviocytes, we found a similar gene expression pattern and response to tofacitinib treatment as reported for keratinocytes. Hence, the tofacitinib-induced downregulation of AVP expression is not a cell-specific phenomenon. Also here, the most dramatic effect is seen for AVPs such as MX1, OAS, and ISG15. We report here that IL-29 significantly increased MX1, OAS2, and ISG15 gene expression in synoviocytes (in the absence of tofacitinib). A role of IL-29 in rheumatoid arthritis [41] and synovial fibroblasts [42] has been reported earlier.

The main classes of AMPs in humans are cathelidins and defensins [18, 19]. AMPs bind to bacterial components such as LPS or LTA. In the further process, these AMPs integrate themselves into the membrane of the bacteria and form holes or prevent the bacteria from division due to the disrupted membrane. AMPs are only secreted in low concentrations in healthy skin as well as healthy synovial membranes but the production is highly upregulated in inflammatory conditions such as psoriasis, rosacea, osteoarthritis, and rheumatoid arthritis [20–24]. In contrast to the expression of AVPs, we found that gene expression of AMPs is only minimally affected by JAK pathway inhibition using tofacitinib. It has been reported by Srivastava et al. that tofacitinib significantly downregulates S100A7, S100A8, and S100A9 in human primary keratinocytes at a concentration as low as 100 nM [43]. This finding is not reproduced in our cohort, but we do see a trend towards downregulation of S100A7 in the presence of both concentrations (100 nM and 600 nM) of tofacitinib. To sum this part up, the antiviral immunity is pronouncedly inhibited by tofacitinib in vitro, while the antimicrobial immunity does not seem to be affected.

There is conflicting data on how JAK inhibition is influencing the development and differentiation of T_H_17 cells. Some researchers believe that JAK inhibition promotes the general differentiation towards T_H_17 cells [44], but others provide data concluding that JAK inhibition prevents differentiation into the pathogenic type of T_H_17 cells [45, 46]. Non-classical or pathogenic human T_H_17 cells have been associated with autoimmune disease [35]. Classical CCR4+CXCR3− T_H_17 cells produce high levels of IL-17 but low levels of IFN-γ. In contrast, non-classical CCR4−CXCR3+ T_H_17 cells express low levels of IL-17 and large amounts of IFN-γ [47]. The presence of more regulated T_H_17 cells may explain the improvements seen under JAK inhibition in different autoimmune diseases. Against this background, we next investigated if the presence of tofacitinib drives naïve CD4^+^ cells rather towards a classical T_H_17 cell phenotype or towards a more pathogenic T_H_17 cell phenotype. Tofacitinib was added at concentrations of 100 nM and 600 nM before the polarizing cytokines TGF-β1, IL-6, and IL-23 as well as the antibody anti-IL4 was added. Looking at the gene expression, we found a slight trend towards RORC gene expression, which is associated with classical (less pathogenic) T_H_17 cells [35]. A similar result can be observed in gene expression of interleukins where the classical T_H_17 cell cytokine IL17A seems to be slightly favoured over IFNG and GMCSF (both more characteristic for pathogenic T_H_17 cells) in the presence of tofacitinib. We furthermore report that IL-17 protein expression is slightly increased in tofacitinib-treated T_H_17 cell cultures, while IL-26, an interleukin with antimicrobial effects, is downregulated. In a study by Ghoreschi et al. [46], it was reported that pathogenic T_H_17 cells differentiated using IL-6 and IL-23 did show less IL-17 and IL-22 secretion under JAK inhibition, while classical T_H_17 cells (differentiated using TGF-β) showed an increased IL-17 secretion. Basically, tofacitinib suppressed pathogenic T_H_17 cells in this study [46]. This is then in line with our data, except that our experimental setup allowed differentiation towards both T_H_17 cell phenotypes at the same time. In contrast, a study in rheumatoid arthritis patients showed that tofacitinib inhibits the secretion of both IL-17 and IFN-γ in anti-CD3/anti-CD28 stimulated CD4^+^ T cells [48].

To investigate the effects of tofacitinib on T cell activation, we stimulated PBMCs with anti-CD2/CD2R antibody or varicella zoster envelope glycoprotein (VZV gE) and analysed the surface expression of CD69 on CD3^+^ T cells via flow cytometry. The surface molecule CD69 is an early activation marker found mainly on lymphocytes and signals via the JAK/STAT pathway [49]. Only recently, the role of CD69 besides being an early activation marker has been started to be investigated. It seems as if CD69 also plays an important role in different immune responses and also T cell differentiation [49]. We show here that CD69 is downregulated in the presence of tofacitinib. The induction of CD69, whether it was by an anti-CD2/CD2R antibody or VZV gE, did not modify the effects of tofacitinib. A downregulation of CD69 in the presence of tofacitinib might negatively impact the immune tolerance as it has been shown that CD69 is required for regulatory T cells [50]. Additionally, the downregulation of CD69 in the presence of VZV gE could possibly explain the increased zoster infections reported in tofacitinib-treated patients [27]. On the other hand, it was shown that CD69 expression is associated with T_H_17 cell differentiation as the lack of CD69 significantly increases IL-17 secretion in CD4^+^ T cells [51]. This is in line with our findings showing an increased gene and protein expression of IL-17 when naïve T cells are polarized towards T_H_17 cells and treated with tofacitinib. We thus hypothesize that the downregulation of CD69 by tofacitinib might be beneficial for differentiation into classical T_H_17 cells. Furthermore, as there is simultaneously a decrease in IFN-γ, it points towards a differentiation of classical T_H_17 cells [51]. This would then be in line with our data suggesting tofacitinib drives naïve CD4^+^ T cells towards a classical T_H_17 cell phenotype rather than a pathogenic phenotype. This favourable development of classical T_H_17 in the presence of tofacitinib partly explains the beneficial effects of the drug in patients [1].

Primary VZV infection induces both, specific antibodies as well as specific T cell-dependent immune responses, respectively [52]. The latter one is necessary to control latent VZV in a subclinical state [52]. In general, type I and II IFNs are important T-cell-derived mediators for antiviral responses. In the case of VZV, T-cell-derived IFN-γ has been shown to be more potent in inhibiting VZV replication when compared to IFN-α [53]. Therefore, we speculate that on the one hand tofacitinib-induced differentiation towards classical T_H_17 cells is followed by a reduction of IFN-γ secretion that is beneficial for patients with autoimmune diseases but on the other hand it facilitates VZV reactivation.

The findings of this study have to be seen in light of some limitations. The presented data and resulting conclusions are based on in vitro experiments. These data correlate with clinical observations but future studies will have to prove if antiviral responses are also negatively affected in patients treated with tofacitinib. Presuming that VZV initiates an infection by invading mucosal epithelia, further analyses of the innate response to VZV antigens by these epithelia (under treatment with tofacitinib) are needed to complete our knowledge about the impact of JAK inhibition in the context of viral, in particular, of VZV infections. Moreover, it remains elusive if other JAK inhibitors impact the antiviral response in the same way as tofacitinib.

## Conclusion

To conclude, we report a strong inhibition of the gene expression of antiviral peptides in both keratinocytes and synoviocytes under tofacitinib treatment in vitro. This observation partially explains the side effects seen under JAK blockade in patients. Furthermore, we report that a JAK inhibition leads to a diminished T cell activation regardless of the activation pathway. These observations shed light onto the antiviral immune mechanisms involved in JAK signalling and partially explain the side effects seen under tofacitinib treatment in patients.

## Supplementary Information

**Additional file 1.**

## Data Availability

The datasets used and/or analysed during the current study are available from the corresponding author on reasonable request.

## Abbreviations

ACK: Ammonium-chloride-potassium erythrocyte lysis buffer
AMP: Antimicrobial peptide
AVP: Antiviral peptide
BST2: Bone marrow stromal cell antigen 2
CAMP: Cathelicidin antimicrobial peptide (also called LL37)
CPT: Cell preparation tubes
DMEM: Dulbecco’s modified Eagle medium
DMSO: Dimethyl sulfoxide
FCS: Foetal calf serum
GATA3: GATA binding protein 3
GMCSF: Granulocyte-macrophage colony-stimulating factor
HFLS: Human fibroblast-like synoviocytes
IFN: Interferon
IL: Interleukin
ISG: Interferon-stimulated gene
JAK: Janus kinase
LPS: Lipopolysaccharides
LTA: Lipoteichoic acids
MTT: 3-(4,5-Dimethylthiazol-2-yl)-2,5-diphenyltetrazolium
OAS2: 2′-5′-Oligoadenylate synthetase 2
PBMC: Peripheral blood mononuclear cell
PBS: Phosphate-buffered saline
PsA: Psoriasis arthritis
RA: Rheumatoid arthritis
RORC: RAR-related orphan receptor C
RPMI: Roswell Park Memorial Institute medium
STAT: Signal transducers and activator of transcription
TBET: T-box transcription factor expressed in T cells
TGF: Transforming growth factor
TNF: Tumour necrosis factor
Tofa: Tofacitinib
VZV gE: Varicella zoster virus envelope glycoprotein E
