# Supplementary material for: Tofacitinib downregulates antiviral immune defence in keratinocytes and reduces T cell activation
Source: Arthritis Res Ther. 2021 May 21;23:144. doi: 10.1186/s13075-021-02509-8 (PMC8138978; doi:10.1186/s13075-021-02509-8)
Supplement: Supplementary file 1 — Additional file 1. [file 13075_2021_2509_MOESM1_ESM.docx]

**Supplemental information**

**Tofacitinib downregulates antiviral immune defence in keratinocytes and reduces T cell activation**

Heike C. Hawerkamp^1^, Alina Domdey^1^, Lisa Radau^1^, Philipp Sewerin^2^, Péter Oláh^1,3^, Bernhard Homey^1^_,_ Stephan Meller^1^

*^1^ Department of Dermatology, Medical Faculty, Heinrich-Heine-University, Duesseldorf, Germany*

*^2^ Department and Hiller Research Unit for Rheumatology, Medical Faculty, Heinrich-Heine-University, Düsseldorf, Germany*

*^3^ Department of Dermatology, Venereology and Oncodermatology, University of Pécs, Pécs, Hungary*

**Supplemental figures**

**
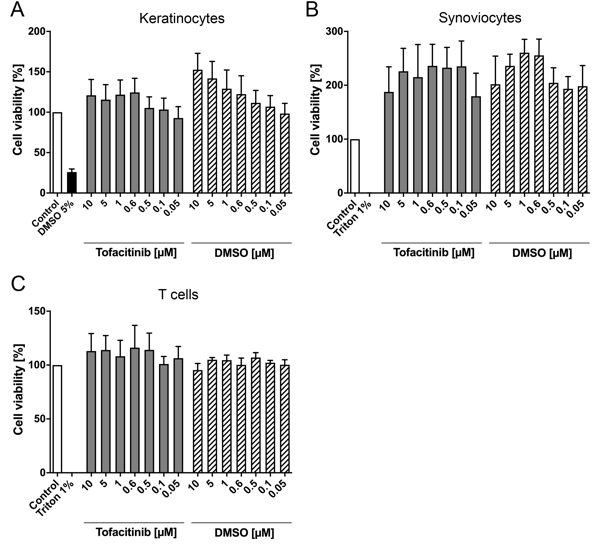
**

SUPPLEMENTAL FIGURE 1: Tofacitinib does not affect cell viability of keratinocytes, synoviocytes and T cells. Keratinocytes (n=5-6, A), synoviocytes (n=3, B) and T cells (n=3, C) were stimulated for 24h with tofacitinib with concentrations ranging from 10 μM to 0.05 μM before a MTT assay (keratinocytes) or CellTiterGlo assay (synoviocytes and T cells) was conducted. For MTT, the OD was measured at 540 nm, while the CellTiterGlo assay is based on luminescence. Viability in untreated control cells was set to 100%.


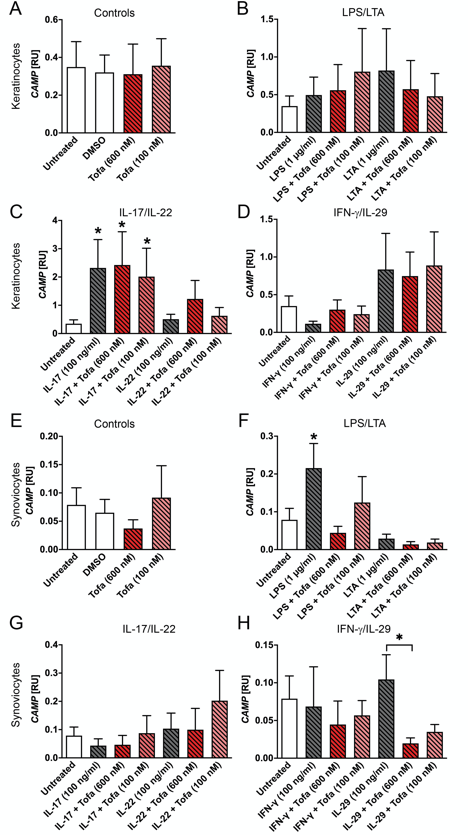


SUPPLEMENTAL FIGURE 2: Tofacitinib does not affect gene expression of antimicrobial CAMP in keratinocytes but downregulates gene expression of IL-29-induced CAMP in synoviocytes. Keratinocytes (n=5-8) or synoviocytes (n=4-10) were stimulated for 60 min with tofacitinib before addition of respective cytokine (IL-17, IL-22, IFN-γ or IL-29) or bacterial component (LPS or LTA). A) CAMP gene expression in keratinocytes, B) CAMP gene expression in synoviocytes, analyzed via qPCR. Statistical analysis was done using Mann-Whitney *U* test and significances are shown as follows: * equals P ≤ 0.05, ** equals P ≤ 0.01 and *** equals P ≤ 0.001.


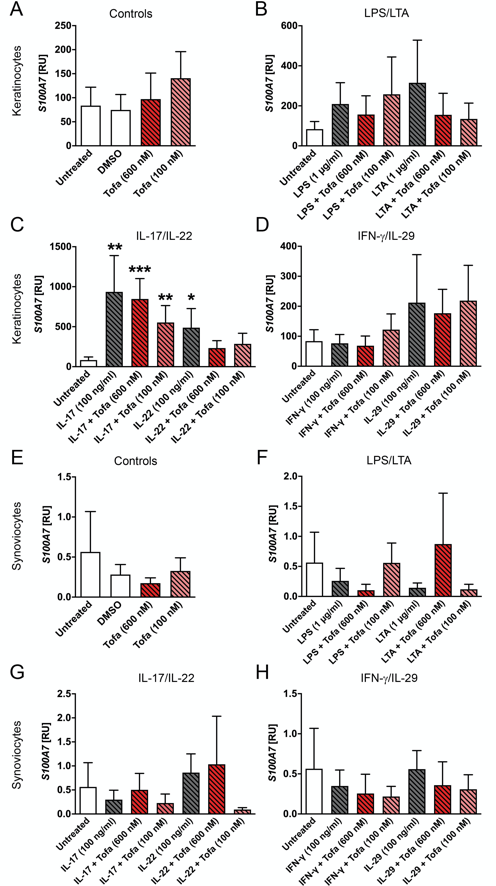


SUPPLEMENTAL FIGURE 3: Tofacitinib (Tofa) does not affect gene expression of antimicrobial psoriasin in keratinocytes and synoviocytes. Keratinocytes (n=5-8) or synoviocytes (n=4-10) were stimulated for 60 min with tofacitinib before addition of respective cytokine (IL-17, IL-22, IFN-γ or IL-29) or bacterial component (LPS or LTA). A) S100A7 gene expression in keratinocytes, B) S100A7 gene expression in synoviocytes, analyzed via qPCR. Statistical analysis was done using Mann-Whitney *U* test and significances are shown as follows: * equals P ≤ 0.05, ** equals P ≤ 0.01 and *** equals P ≤ 0.001.


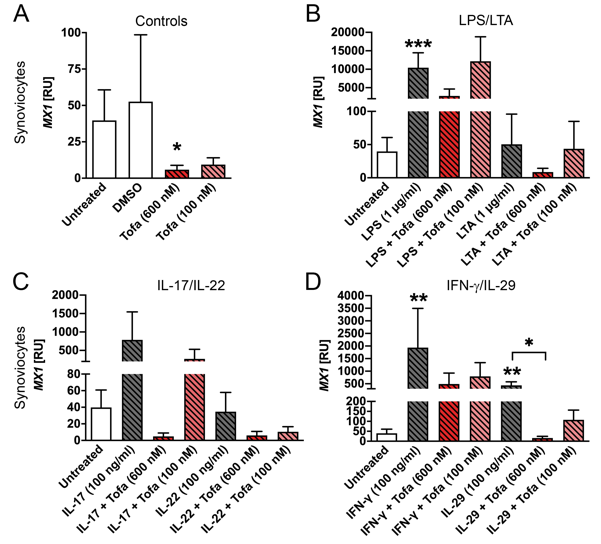


SUPPLEMENTAL FIGURE 4: Tofacitinib downregulates gene expression of MX1 at baseline or in presence of IFN-γ or IL-29. Synoviocytes (n=4-10) were stimulated for 60 min with tofacitinib before addition of respective cytokine or bacterial component. MX gene expression is shown for controls (A), tofacitinib in combination with LPS or LTA (B), in combination with IL-17 or IL-22 (C) or tofacitinib combined with IFN-γ or IL-29 (D). The cell lysates were analysed via qPCR. Statistical calculation was done using Mann-Whitney *U* test. Significances were compared to untreated control are depicted directly above the stimulatory agent or the compared conditions are indicated by lines and depicted as follows: * equals P ≤ 0.05, ** equals P ≤ 0.01 and *** equals P ≤ 0.001.


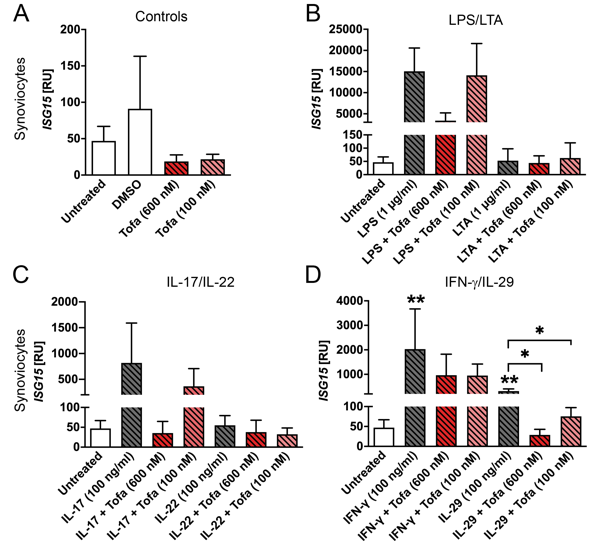


SUPPLEMENTAL FIGURE 5: Tofacitinib alters ISG15 gene expression at baseline level. Synoviocytes (n=4-10) were stimulated for 60 min with tofacitinib before addition of respective cytokine (IL-17, IL-22, IFN-γ or IL-29) or bacterial component (LPS or LTA). The cell lysates were analysed via qPCR. Statistical calculation was done using Mann-Whitney *U* test. Significances were compared to untreated control are depicted directly above the stimulatory agent or the compared conditions are indicated by lines and depicted as follows: * equals P ≤ 0.05, ** equals P ≤ 0.01 and *** equals P ≤ 0.001.


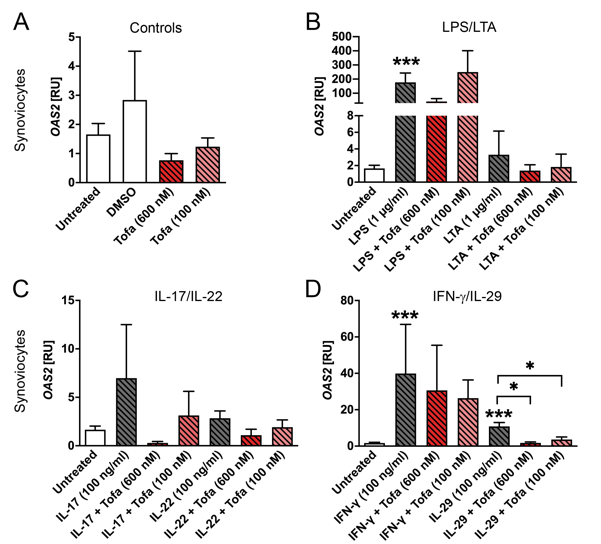


SUPPLEMENTAL FIGURE 5: Antiviral OAS2 gene expression is downregulated by tofacitinib. Synoviocytes (n=4-10) were stimulated for 60 min with tofacitinib before addition of respective cytokine or bacterial component. OAS2 gene expression is shown for controls (A), tofacitinib in combination with LPS or LTA (B), in combination with IL-17 or IL-22 (C) or tofacitinib combined with IFN-γ or IL-29 (D). The gene expression was analysed via qPCR. Statistical analysis was done using Mann-Whitney *U* test. Significances were compared to untreated control are depicted directly above the stimulatory agent or the compared conditions are indicated by lines and depicted as follows: * equals P ≤ 0.05, and *** equals P ≤ 0.001.

**
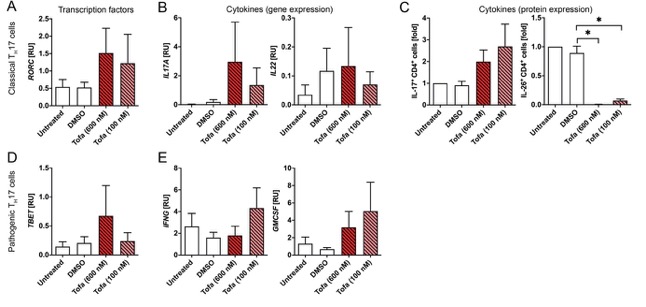
**

SUPPLEMENTAL FIGURE 7: Tofacitinib slightly upregulates RORC and inhibits IL-26 protein expression. Naïve CD4^+^ T cells (n=5-6) were polarized for 7 days towards T_H_17 cells in presence or absence of tofacitinib at the indicated concentrations. The cells were harvested, RNA isolated and used for qPCR analysis. Displayed are gene expressions of RORC, IL17A and IL22 (A/B; indicative of classical of T_H_17 cells) and TBET, IFNG and GMCSF (D, E; indicators of pathogenic T_H_17 cells). A proportion of cells were stained with antibodies against CD4 as well as against IL-17 and IL-26 (C) and analyzed via flow cytometry. Mann Whitney *U* test and significances are shown compared to DMSO control and indicated as * equals P ≤ 0.05.
